# Left-sided appendicitis due to anatomical variation: A case report

**DOI:** 10.3389/fsurg.2022.896116

**Published:** 2022-08-24

**Authors:** Qiang Hu, Jianfeng Shi, Yuanshui Sun

**Affiliations:** Department of General Surgery, Tongde Hospital of Zhejiang Province, Hangzhou, China

**Keywords:** intestinal malrotation, left side, abdominal pain, acute appendicitis, case report

## Abstract

**Introduction:**

Left-sided appendicitis is a rare condition, and we report a patient with left abdominal heterotopia of the right colon complicated by acute appendicitis in the left lower quadrant.

**Case presentation:**

A 39-year-old male was admitted to hospital following left lower abdominal pain for 1 day. Imaging examination by abdominal CT showed that the appendix was not clearly seen, and a mass was found in the left lower abdomen. Because the patient's abdominal pain was severe and the current diagnosis was not clear, after soliciting the patient's consent, we performed laparoscopic exploration. This exploration revealed that the cecum and ascending colon were located in the left iliac fossa, the appendix was swollen, the length of the appendix was approximately 6 cm, the diameter of the appendix was approximately 1 cm, and there was pus moss attached to the surface. We performed a laparoscopic appendectomy; the procedure was uneventful and the patient was discharged 3 days after the procedure.

**Conclusion:**

Left-sided appendicitis is a rare condition and is therefore easy to misdiagnose. Wrong diagnosis can lead to serious complications and endanger the patient's life. Therefore, a full combination of laboratory tests and CT scan is required. If still no diagnosis can be made correctly, a laparoscopic exploration needs to be performed in a timely manner. This case teaches us that when we encounter a patient with severe left abdominal pain that cannot be definitely diagnosed, we need to be vigilant and perform timely laparoscopic exploration when necessary.

## Introduction

Intestinal malrotation is attributed to an abnormal development of the midgut during the embryonic period. When the ascending colon rotates in the opposite direction, the appendix is ectopic in the left lower quadrant. When the appendix has inflammation, and if it is not treated on time, the appendix suppurates and perforates, which easily causes periappendiceal abscess, peritonitis, and septic shock, and becomes life-threatening in severe cases (1). Ectopic appendicitis has varied symptoms and signs due to its location variation, which often brings great difficulties to clinical diagnosis and treatment. Therefore, doctors may misjudge the patient's condition.

## Case presentation

**Figure 1:**
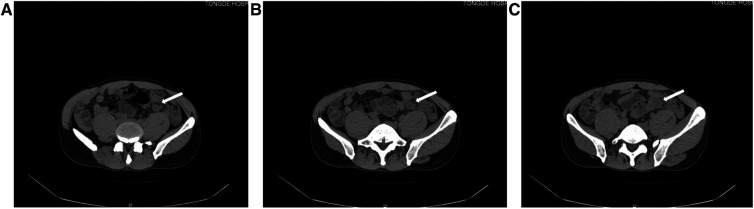
The appendix is not clearly visualized, and a mass can be seen in the left abdomen.

**Figure 2:**
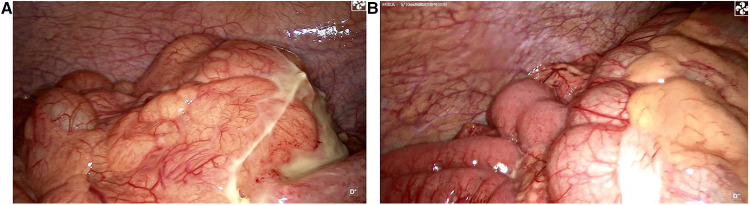
(**A**) Laparoscopic exploration reveals an omentum-enclosed mass with pus moss on the surface at the left iliac fossa of the patient. (**B**) Right hemicolon and cecum, the right iliac fossa is occupied by the ileum and it pushes the cecum and ascending colon to the left iliac fossa.

**Figure 3:**
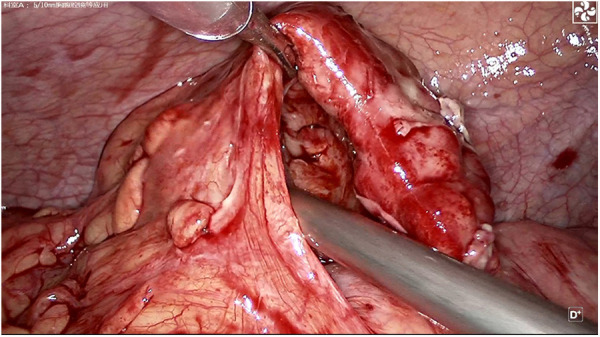
Intraoperative appendix.

**Figure 4:**
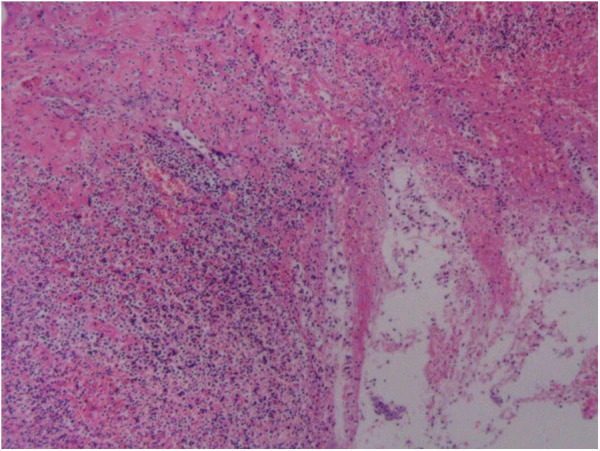
Postoperative pathology reveals acute gangrenous appendicitis with periappendicitis (HE × 100).

A 39-year-old male patient was admitted to hospital due to “left lower abdominal pain for 1 day.” On physical examination, T was 37.3°C, P 78 beats per minute, R 18 breaths per minute, and BP 115/61 mmHg. There were no obvious abdominal bulge, tenderness, and rebound pain in the left lower abdomen, bowel sounds were 4 times/min, and there was no shifting dullness. Laboratory tests showed that the white blood cell count was 10.2 × 10^9^/L, neutrophils was 91.4%, lymphocytes (%) 6.0%, and C-reactive protein 301.2 mg/L. Imaging examination by abdominal CT showed that the appendix was not clearly visible, and a mass was found in the left lower abdomen (Figure 1). Because the patient's abdominal pain was severe and the current diagnosis was not clear, after soliciting the patient's consent, we performed laparoscopic exploration. It revealed an omentum-enclosed mass with pus moss on the surface at the left iliac fossa of the patient (Figure 2A). Laparoscopic exploration revealed the right iliac fossa occupied by ileum that pushed the cecum and ascending colon to the left iliac fossa, which is an intestinal malrotation with a left-sided appendicitis (Figure 2B), and intestinal malrotation left-sided appendicitis was diagnosed intraoperatively. The pus on the surface of the mass was aspirated and the omentum was detached by blunt dissection with the aspirator to expose the appendix, which was seen to be swollen, approximately 6 cm in length, approximately 1 cm in diameter, and with pus moss attached to the surface (Figure 3). Subsequently, we performed laparoscopic appendectomy, and the procedure was uneventful, detailed surgical video can be found in the supplementary material. Antibiotics were used after the operation. On the first postoperative day, the patient had flatus in the anus but without defecation and was given liquid diet. On the second postoperative day, the patient had defecation and was given a semifluid diet. A re-examination of the blood routine 3 days after operation showed WBC: 5.1 × 10^9^/L, NEU: 61%, LYM: 6.0%, CRP: 3.0 mg/L. Since the patient's blood routine examination was normal on the third day without fever, we stopped using antibiotics and the patient was discharged. Postoperative pathology revealed acute gangrenous appendicitis with periappendicitis (Figure 4), and on telephone follow-up on the seventh postoperative day, the patient reported good recovery without any complications.

## Discussion

Left-sided appendicitis is a rare digestive system disease requiring emergency surgery, is classified according to congenital anatomical abnormalities, mainly situs inversus and intestinal malrotation, and accounts for approximately 93.6% of cases (2). Due to the abnormal anatomy of the left appendix, and many times without typical clinical manifestations, it is easy to misdiagnose. Once the diagnosis is delayed, it may lead to periappendiceal abscess or perforation, which may be life-threatening in severe cases.

Congenital abdominal heterotaxy, with the ascending colon located in the left abdomen and the descending and sigmoid colon located in the right abdomen, is often accompanied by heterotopia of other organs such as the heart and liver, and the cecum is also located in the left lower abdomen (3). However, this patient is different from the one with situs inversus, in the sense that the cecum and ascending colon are still on the left side of the abdominal cavity and the descending colon is also on the left side during embryonic development, when the small intestine is located on the right side of the right colon and the appendix is also far from the right lower quadrant (4).

The symptoms and signs of ectopic appendicitis can vary due to the variation of location, which often brings considerable difficulties to clinical diagnosis and treatment. Ectopic appendix in the left lower quadrant is rare, acute appendicitis is easily misdiagnosed as a sigmoid colon, and urinary system diseases and gynecological diseases occur in female patients (5). Therefore, doctors may misjudge the patient's condition, resulting in the loss of the best operation opportunity, which results in complications. When clinicians encounter patients with left lower abdominal pain during practice, they should think divergently to consider the possibility of this pain leading to disease, while excluding the possibility of other diseases, so as not to misdiagnose and mistreat.

To make a correct diagnosis of ectopic appendicitis at an early stage requires surgeons to have rich clinical experience and be familiar with the type and clinical characteristics of ectopic appendicitis. Physicians need to correctly understand the characteristics of severe abdominal pain in appendicitis and its diagnostic value in appendicitis. If necessary, severe pain, combined with imaging examinations and white blood cell count and C-reactive protein test results, to the exclusion of other diseases, can suggest the diagnosis and facilitate improvement in the correct diagnosis rate and reduce misdiagnosis (6). In particular, CT, abdominal ultrasonography, laparoscopy, and CRP are of great value in the diagnosis and differential diagnosis of acute appendicitis (7). Rao et al. retrospectively analyzed 209 cases of appendectomy and concluded that the coincidence rate between the results of abdominal CT examination and postoperative pathological diagnosis was as high as 93%, and for non-appendicitis patients with suspected appendicitis, the exclusion rate of abdominal CT was 99% (8). Detection of CRP in peripheral blood by Pruekprasert et al revealed its high sensitivity and accuracy for the diagnosis of acute appendicitis (9).

Once ectopic appendicitis is diagnosed, surgical treatment should be given actively, and for a small number of patients with suspected appendicitis, it is indeed difficult to confirm the diagnosis, and if available, laparoscopic exploration is feasible. Under the laparoscope, the abdominal cavity and pelvic cavity can be explored to determine the diagnosis of ectopic appendicitis and exclude acute abdomen caused by other diseases. Laparoscopic appendectomy can reduce the incidence of incision infection and abdominal abscess. It has the advantages of less trauma, faster postoperative recovery, and fewer complications (10). Therefore, it is a safe and effective method for the treatment of ectopic appendicitis.

## Conclusion

Left-sided appendicitis is a rare condition and is therefore easy to misdiagnose. Wrong diagnosis can lead to serious complications and endanger the patient's life. Therefore, a full combination of laboratory tests and CT scan is required. If still no diagnosis can be made, a laparoscopic exploration needs to be performed in a timely manner. This case teaches us that when we encounter a patient with severe left abdominal pain that cannot be definitely diagnosed, we need to be vigilant and perform timely laparoscopic exploration when necessary.

## Data Availability

The original contributions presented in the study are included in the article/**Supplementary Material**, Further inquiries can be directed to the corresponding author/s.

## References

[1] Chuang PW Huang BM Liu CH Chen CC Tsai MJ. Left-sided appendicitis in an elderly patient with midgut malrotation. Indian J Surg. (2015) 77:1418–20. 10.1007/s12262-014-1200-9 27011586 PMC4775601

[2] Shimamura Y Nishiyama T Taketa T Fujita Y. Education and imaging. Gastroenterology: acute left-sided appendicitis with intestinal malrotation. J Gastroenterol Hepatol. (2015) 30:1446. 10.1111/jgh.12969 26361360

[3] Hollander SC Springer SA. The diagnosis of acute left-sided appendicitis with computed tomography. Pediatr Radiol. (2003) 33:70–1. 10.1007/s00247-002-0829-x 12497246

[4] Mushtaq Z Sarwar MZ Ya R Naqi SA. Left sided appendicitis—sa surgical dilemma: case report. J Pak Med Assoc. (2021) 71:1483–5. 10.47391/JPMA.09-1108 34091640

[5] Chick JFB Chauhan NR Mullen KM Hanna JW Bair RJ Khurana B. Intestinal malrotation and acute left-sided appendicitis. J Emerg Med. (2013) 44:e333–4. 10.1016/j.jemermed.2012.11.053 23419212

[6] Akbulut S Ulku A Senol A Tas M Yagmur Y. Left-sided appendicitis: review of 95 published cases and a case report. World J Gastroenterol. (2010) 16:5598–602. 10.3748/wjg.v16.i44.5598 21105193 PMC2992678

[7] Seifmanesh H Jamshidi K Kordjamshidi A Delpisheh A Peyman H Yasemi M. Acute left-sided appendicitis with situs inversus totalis: a case report. Am J Emerg Med. (2010) 28:1058.e5–e7. 10.1016/j.ajem.2010.01.020 20825854

[8] Rao PM Rhea JT Rattner DW Venus LG Novelline RA. Introduction of appendiceal CT: impact on negative appendectomy and appendiceal perforation rates. Ann Surg. (1999) 229:344–9. 10.1097/00000658-199903000-00007 10077046 PMC1191699

[9] Pruekprasert P Maipang T Geater A Apakupakul N Ksuntigij P. Accuracy in diagnosis of acute appendicitis by comparing serum C-reactive protein measurements, Alvarado score and clinical impression of surgeons. J Med Assoc Thai. (2004) 87:296–303. 15117047

[10] Lin HF Lai HS Lai IR. Laparoscopic treatment of perforated appendicitis. World J Gastroenterol. (2014) 20:14338–47. 10.3748/wjg.v20.i39.14338 25339821 PMC4202363

